# Mesenchymal stem cells-derived small extracellular vesicles alleviate diabetic retinopathy by delivering NEDD4

**DOI:** 10.1186/s13287-022-02983-0

**Published:** 2022-07-15

**Authors:** Fengtian Sun, Yuntong Sun, Junyan Zhu, Xiaoling Wang, Cheng Ji, Jiahui Zhang, Shenyuan Chen, Yifan Yu, Wenrong Xu, Hui Qian

**Affiliations:** Jiangsu Key Laboratory of Medical Science and Laboratory Medicine, School of Medicine, Jiangsu University, Zhenjiang, 212013 Jiangsu China

**Keywords:** MSC-sEV, NEDD4, DR, RPE, PTEN, AKT, NRF2

## Abstract

**Background:**

As a leading cause of vision decline and severe blindness in adults, diabetic retinopathy (DR) is characterized by the aggravation of retinal oxidative stress and apoptosis in the early stage. Emerging studies reveal that mesenchymal stem cells-derived small extracellular vesicles (MSC-sEV) treatment represents a promising cell-free approach to alleviate ocular disorders. However, the repairing effects of MSC-sEV in DR remain largely unclear. This study aimed at exploring the role and the underlying mechanism of MSC-sEV in hyperglycemia-induced retinal degeneration.

**Methods:**

In vivo, we used streptozotocin (STZ) to establish diabetic rat model, followed by the intravitreal injection of MSC-sEV to determine the curative effect. The cell viability and antioxidant capacity of retinal pigment epithelium (RPE) cells stimulated with high-glucose (HG) medium after MSC-sEV treatment were analyzed in vitro. By detecting the response of cell signaling pathways in MSC-sEV-treated RPE cells, we explored the functional mechanism of MSC-sEV. Mass spectrometry was performed to reveal the bioactive protein which mediated the role of MSC-sEV.

**Results:**

The intravitreal injection of MSC-sEV elicited antioxidant effects and counteracted retinal apoptosis in STZ-induced DR rat model. MSC-sEV treatment also reduced the oxidative level and enhanced the proliferation ability of RPE cells cultured in HG conditions in vitro. Further studies showed that the increased level of phosphatase and tensin homolog (PTEN) inhibited AKT phosphorylation and nuclear factor erythroid 2-related factor 2 (NRF2) expression in RPE cells stimulated with HG medium, which could be reversed by MSC-sEV intervention. Through mass spectrometry, we illustrated that MSC-sEV-delivered neuronal precursor cell-expressed developmentally downregulated 4 (NEDD4) could cause PTEN ubiquitination and degradation, activate AKT signaling and upregulate NRF2 level to prevent DR progress. Moreover, NEDD4 knockdown impaired MSC-sEV-mediated retinal therapeutic effects.

**Conclusions:**

Our findings indicated that MSC-sEV ameliorated DR through NEDD4-induced regulation on PTEN/AKT/NRF2 signaling pathway, thus revealing the efficiency and mechanism of MSC-sEV-based retinal protection and providing new insights into the treatment of DR.

**Supplementary Information:**

The online version contains supplementary material available at 10.1186/s13287-022-02983-0.

## Background

Diabetic retinopathy (DR) is one of the common complications of diabetes mellitus [1]. Hyperglycemia microenvironment induced the generation of reactive oxygen species (ROS) promotes the redox imbalance and DNA damage of retinal cells [2, 3]. Due to the critical roles of RPE cells in maintaining the retinal function and structure including nutrients transmission, retinal leakage prevention and photoreceptors protection, increased RPE cells loss in response to the aggravation of oxidative stress is recognized as an essential risk factor of DR in the early stage [4–6]. Currently, DR has been considered a leading cause of vision decline and blindness in adults worldwide because of the limited treatments [7]. Therefore, there is an urgent need to strengthen the understanding of the mechanism for DR progress and develop an alternative therapy to improve retinal injury.

Mesenchymal stem cells (MSCs) transplantation represents a promising therapeutic strategy for tissue regeneration with the superiority of low immunogenicity, excellent differentiation ability and convenient isolation [8]. Recent evidence reveals that MSCs exert repairing effects mainly through the paracrine pathway [9]. As the main component of membranous vesicles secreted by living cells, small extracellular vesicles (sEV) carrying various functional cargos mediate cell-to-cell communication by regulating recipient cells activity [10, 11]. Our researches have previously confirmed the therapeutic value of human umbilical cord MSCs-derived sEV (MSC-sEV) in type 2 diabetes, renal fibrosis and cutaneous wound healing [12–14]. Importantly, increasing studies suggest that MSC-sEV treatment can be served as a novel cell-free therapy approach for ocular diseases including glaucoma, subretinal fibrosis and retinal ischemia [15–17]. However, whether MSC-sEV administration can prevent DR progress still needs further investigations.

In this study, we explored the role of human umbilical cord MSC-sEV in retinal degeneration by establishing streptozotocin (STZ)-induced DR rat model. We found that MSC-sEV injection ameliorated retinal apoptosis and oxidative stress to alleviate DR progress. In vitro, MSC-sEV administration promoted RPE cells proliferation and enhanced their antioxidant ability under high-glucose (HG) conditions. Mechanically, MSC-sEV-delivered neuronal precursor cell-expressed developmentally downregulated 4 (NEDD4) promoted phosphatase and tensin homolog (PTEN) ubiquitination and activated AKT/NRF2 signaling, thus improving retinal injury. These findings present a new mechanism of MSC-sEV-induced retinal therapeutic effects and provide a promising treatment approach for DR.

## Methods

### Ethics

This study was approved by the Medical Ethics Committee and Ethics Committee for Experimental Animals of Jiangsu University (2,020,161).

### Cell culture and identification

MSCs were isolated from fresh umbilical cords which were collected from mothers at the Affiliated Hospital of Jiangsu University after obtaining their agreement. Briefly, the cords were washed with phosphate-buffered saline (PBS) containing penicillin and streptomycin to remove the cord blood, cut into 1-mm^2^-sized pieces and maintained in minimal essential medium alpha (α-MEM; Invitrogen, Grand Island, NY, USA) containing 15% fetal bovine serum (FBS; Gibco, Grand Island, USA) at 37 °C with 5% CO_2_. The medium was replaced every 3 days. After 10 days, the fibroblast-liked cells were trypsinised and passaged for further expansion. Subsequently, MSCs were cultured in α-MEM (Invitrogen, Grand Island, NY, USA) containing 10% FBS (Gibco, Grand Island, USA) at 37 °C with 5% CO_2_. MSCs in passage 3 were cultured in osteogenic medium (Cyagen Biosciences, CA, USA) or adipogenic medium (Cyagen Biosciences, CA, USA) for 2 weeks and then stained with Alizarin Red or Oil Red O to detect their multidifferentiation ability. Flow cytometry assay was performed to detect the surface markers including CD29, CD44, CD166, CD45, HLA-DR and CD14.

Human fetal lung fibroblast 1 (HFL1) and RPE cells were purchased from the cell bank of the Chinese Academy of Sciences and maintained in low-glucose (LG) Dulbecco’s modified Eagle’s medium (DMEM; Invitrogen, Grand Island, NY, USA) with 10% FBS (Gibco, Grand Island, USA) at 37 °C with 5% CO_2_.

### Isolation and identification of sEV

The cell supernatants of MSCs or HFL1 cultured in serum-free DMEM for 48 h were collected and centrifuged at 300 g for 5 min, 2,000 g for 10 min and 10,000 g for 30 min to remove cells and cell debris. Subsequently, MSC-sEV or HFL1-derived sEV (HFL1-sEV) were precipitated after ultracentrifugation at 100,000 g for 3 h and washed with PBS. After filtration using 0.22-μm filter, purified sEV were obtained and stored at -80 °C. The morphology of sEV was detected by transmission electron microscopy (TEM; FEI Tecnai 12, Philips, Netherlands). The size distribution and particle number of sEV were measured by nanoparticle tracking analysis (NTA; NanoSight, Amesbury, UK). Western blot was used for analyzing the surface markers of sEV including CD9, CD63, heat shock protein 70 (HSP70), Alix, tumor susceptibility gene 101 (TSG101) and Calnexin.

### MSC-sEV labeling and internalization

MSC-sEV were mixed with PKH-26 (Sigma-Aldrich, St. Louis, USA) at 37 °C for 1 h and then washed with PBS to remove the unconjugated dye. RPE cells were seeded into 12-well plates and treated with PKH-26-labeled MSC-sEV for 24 h at 37 °C with 5% CO_2_. After the fixation with 4% paraformaldehyde for 30 min, the cells were stained with Hoechst 33,342 (Sigma-Aldrich, St. Louis, USA) to label nuclei. The internalization of MSC-sEV by RPE cells was observed using a confocal microscope (DeltaVision Elite, GE, USA).

### Animal model and treatment

Sprague–Dawley male rats aged 8 weeks (200 ± 20 g) were purchased from the Animal Centre of Jiangsu University. The rats were housed in a 12/12 h light/dark cycle, at the temperature of 25 °C and the relative humidity of 50% with sufficient food and water. For diabetic model establishment, rats were fed with 45% high-fat diet for 4 weeks and then injected with STZ (35 mg/kg in 0.1 M citrate-buffered saline, pH 4.5; Sigma-Aldrich, St. Louis, USA) via tail vein. Three days later, the rats with fasting blood glucose level above 16.7 mM were considered diabetic rats. Normal rats were fed with normal diets and injected with citrate-buffered saline (0.1 M, pH 4.5) via tail vein. Twelve weeks after STZ injection, diabetic rats were randomly divided into 3 groups and respectively treated with PBS (3 μL), MSC-sEV (1 × 10^6^ particles suspended in 3 μL of PBS) or HFL1-sEV (1 × 10^6^ particles suspended in 3 μL of PBS) by intravitreal injection. Normal rats were injected with PBS (3 μL) intravitreally. Twenty weeks after STZ injection, all rats were killed and the eyeballs of each rat were collected for further analysis.

### Electroretinography (ERG) analysis

Twenty weeks after STZ injection, rats were dark-adapted overnight and anesthetized by the intraperitoneal injection of 30 mg/kg sodium pentobarbital. Under dim red light conditions, bilateral corneal electrodes were placed on the corneal surface, reference electrodes were located in the mouth, and ground electrodes were attached at the tail. White flashes with the intensity of 3.0 cd s/m^2^ were used to induce the response. The UTAS Visual Diagnostic System (LKC Technologies, USA) was applied to record the scotopic a/b-wave.

### Hematoxylin and eosin (H&E) staining and immunohistochemistry staining

Retinal tissues were carefully isolated, fixed in 4% paraformaldehyde overnight, embedded in paraffin and prepared into 4-μm sections. The sections were stained with H&E and observed under a microscope (Nikon, Tokyo, Japan). For immunohistochemistry staining, the paraffin-embedded retinal tissue sections were dewaxed and exposed to 3% hydrogen peroxide for 20 min to inhibit endogenous peroxidase activity. After socking in boiled citrate buffer (pH 6.0, 10 mM) for 30 min to repair antigen activity, the sections were blocked with 5% bovine serum albumin (BSA) for 1 h and incubated with the primary antibody at 4 °C overnight. Subsequently, the sections were washed with PBS, incubated with secondary antibody for 1 h at 37 °C, visualized using diaminobenzidine, counterstained with hematoxylin and observed under a microscope (Nikon, Tokyo, Japan). The primary antibodies included cleaved caspase-3 (1:100, CST, 9661), NRF2 (1:100, Bioworld, BS1258), PTEN (1:100, Bioworld, BS1305), p-AKT (1:100, CST, 4060) and NEDD4 (1:100, Proteintech, 21698-1-AP).

### Quantitative reverse transcription PCR (qRT-PCR)

Total RNA of retinal tissues was isolated using Trizol reagent (Gibco, Grand Island, USA), followed by the reverse transcription into cDNA according to the manufacturer's protocol (Vazyme, Nanjing, China). QRT-PCR was performed to detect the gene expressions by using the SYBR Green PCR kit (Vazyme, Nanjing, China). The primer sequences are listed in Table 1.

**Table 1:** Primer sequences for qRT-PCR

Gene	Sequence (5’-3’)	*T*_*m*_ (°C)	Size (bp)
Rat-β-actin	F: GACCTGTACGCCAACACAGT R: CTCAGGAGGAGCAATGATCT	59	129
Rat-GPX1	F: AATCAGTTCGGACATCAGGAG R: GAAGGTAAAGAGCGGGTGAG	55	150
Rat-HO-1	F: CTGCTAGCCTGGTTCAAGATACT R: TAAATTCCCACTGCCACGGT	58	142
Rat-NQO1	F: TGGCCAATTCAGAGTGGCATT R: AGAGTGGTGACTCCTCCCAG	60	157
Rat-GCLC	F: GCCGTCTTACAGGGGATGTT R: ACGCCTTCCTTCCCATTGAT	58	158
Rat-GCLM	F: GTGGGCACAGGTAAAACCCAA R: ACTTGCCTCAGAGAGCAGTTC	60	145

### Western blot

RIPA lysis buffer (Thermo Fisher Scientific, MA, USA) was used to isolate total protein from sEV, retinal tissues or RPE cells. Equal amounts of protein samples were separated by 12% sodium dodecyl sulfate-polyacrylamide gel electrophoresis, transferred to the polyvinylidene fluoride membrane (Millipore, Billerica, MA, USA), blocked in 5% skim milk for 1 h and then incubated with primary antibodies at 4 °C overnight. Subsequently, the membranes were incubated with horseradish peroxidase-conjugated secondary antibodies (Invitrogen, USA) at 37 °C for 2 h and visualized by enhanced chemiluminescence. The primary antibodies are listed in Table 2.

**Table 2:** Primary antibodies for western blot

Antibody	Brand	Number	Dilution ratio
CD9	Abcam	ab236630	1:1000
CD63	Abcam	ab271286	1:1000
HSP70	CST	4873	1:1000
TSG101	Abcam	ab125011	1:1000
Alix	CST	2171	1:1000
Calnexin	CST	2679	1:1000
β-actin	CST	4970	1:5000
PCNA	CST	13,110	1:1000
Bcl-2	Abcam	ab196495	1:1000
Bax	CST	2772	1:1000
NRF2	Bioworld	BS1258	1:1000
HO-1	Proteintech	10701-1-AP	1:1000
NQO1	Proteintech	67240-1-Ig	1:1000
GPX1	Bioworld	BS61511	1:1000
p-AKT	CST	4060	1:1000
AKT	CST	9272	1:1000
p-mTOR	CST	5536	1:1000
mTOR	CST	2972	1:1000
p-ERK	CST	4370	1:1000
ERK	CST	4695	1:1000
p-p65	CST	3033	1:1000
p65	CST	8242	1:1000
p-p38	CST	4511	1:1000
p38	CST	8690	1:1000
p-STAT3	CST	9145	1:1000
STAT3	CST	4904	1:1000
PTEN	Bioworld	BS1305	1:1000
NEDD4	Proteintech	21698-1-AP	1:1000
Ubiquitin	CST	91112	1:1000

### Treatment of RPE cells in vitro

RPE cells were seed into 6-well plates at a density of 2 × 10^5^ cells/well and stimulated with HG DMEM (30 mM) for 48 h to mimic diabetic conditions, followed by the treatment with MSC-sEV (1 × 10^7^ particles suspended in 30 μL of HG DMEM) or HFL1-sEV (1 × 10^7^ particles suspended in 30 μL of HG DMEM) for 24 h. RPE cells treated with 30 μL of HG DMEM were used as control group.

### Cell transfection

MSCs and RPE cells were seed into 6-well plates at a density of 2 × 10^5^ cells/well and cultured in 37 °C incubator overnight. Cells were transfected with siRNA or shRNA by using Lipofectamine 2000 (Life Technologies, Carlsbad, CA, USA) in serum-free medium. After 6 h, cells were cultured in complete medium containing 10% FBS (Gibco, Grand Island, USA). The sequences of siRNA and shRNA are shown in Table 3.

**Table 3:** The sequences of siRNA and shRNA

Target	Sequence (5’-3’)
PTEN siRNA	CCACCACAGCUAGAACUUATT
SiRNA NC	TTCTCCGAACGTGTCACGT
NEDD4 shRNA	CCGGGCTGAACTATACGGTTCAAATCTCGAGATTTGAACCGTATAGTTCAGCTTTTTG
Control shRNA	CCGGGCAAGCTGACCCTGAAGTTCATCTCGAGATGAACTTCAGGGTCACGTTGCTTTTTG

### Cell counting kit-8 (CCK8) assay

The treated RPE cells were seeded into 96-well plate at a density of 3000 cells/well. After 24, 48, 72 and 96 h incubation, each well was exposed to 100 μL of fresh medium containing 10 μL of CCK8 (Vazyme, Nanjing, China) for 3 h. The absorbance at 450 nm was measured by a microplate reader (FLx800, BioTek, Winooski, VT, USA).

### ROS measurement

The treated RPE cells were incubated with 10 μM of 2′-7′-dichlorofluorescein diacetate (Beyotime, Shanghai, China) for 30 min at 37 °C and washed with PBS. The ROS secreted by RPE cells promoted the production of 2′-7′-dichlorofluorescein which is a fluorescent compound. The intracellular fluorescence was observed by a Fluorescent Microscope (DP73, Olympus, Tokyo, Japan).

### Immunofluorescence staining

The treated RPE cells were fixed in 4% paraformaldehyde for 30 min, permeabilized with 0.1% Triton-X100 for 10 min, blocked with 5% BSA for 1 h and incubated with the primary antibody at 4 °C overnight. After washing with PBS, the sections were stained with FITC or Cy3-conjugated goat-anti-rabbit secondary antibody (Sigma-Aldrich, St. Louis, USA) for 1 h at 37 °C, counterstained with Hoechst 33,342 (Sigma-Aldrich, St. Louis, USA) and observed under a confocal microscope (DeltaVision Elite, GE, USA). The primary antibodies included Ki-67 (1:100, CST, 9449), NRF2 (1:100, Bioworld, BS1258), PTEN (1:100, Bioworld, BS1305), p-AKT (1:100, CST, 4060) and NEDD4 (1:100, Proteintech, 21,698–1-AP).

### Co-Immunoprecipitation (co-IP) assay

RPE cells were pretreated with MG132 for 5 h before co-IP assay. Cells were washed with PBS and lysed in co-IP buffer, followed by the incubation with NEDD4 antibody (1:100) at 4 °C overnight. Then, the immunocomplexes were incubated with Protein A/G gel for 3 h at 4 °C and detected by western blot.

### Statistical analysis

All data were shown as means ± SEM and analyzed using GraphPad Prism software (GraphPad, San Diego, USA). The significant differences between two groups were assessed by Student’s t test. The significant differences among multiple groups were assessed by analysis of variance with Newman–Keuls test. *P* value < 0.05 was considered statistically significant.

## Results

### The characteristics of MSCs and sEV

**Fig. 1:**
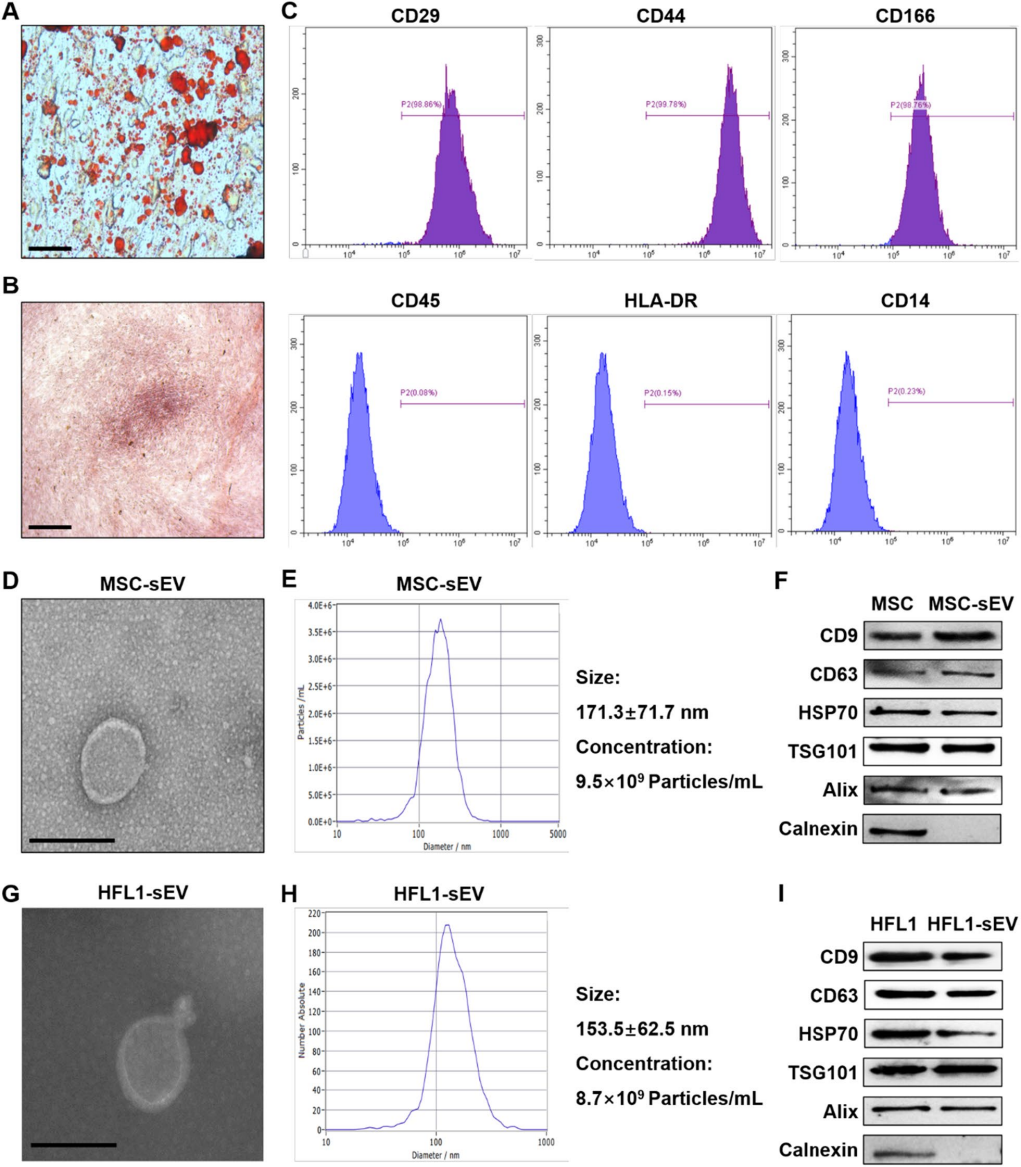
Identification of MSCs and sEV. **A** Oil Red O staining for analyzing the adipogenic differentiation of MSCs. Scale bars, 100 μm. **B** Alizarin Red staining for detecting the osteogenic differentiation of MSCs. Scale bars, 100 μm. **C** Flow cytometry assay for determining the phenotypic markers of MSCs. **D** The morphology of MSC-sEV was identified by TEM. Scale bars, 200 nm. **E** The size distribution and concentration of MSC-sEV were identified by NTA. **F** Western blot for the protein markers of MSC-sEV. **G** The morphology of HFL1-sEV was identified by TEM. Scale bars, 200 nm. **H** The size distribution and concentration of HFL1-sEV were identified by NTA. **I** Western blot for the protein markers of HFL1-sEV

MSCs were isolated from human umbilical cord and exhibited positive staining after treating with Oil Red O and Alizarin Red (Fig. 1A, B). Moreover, flow cytometry assay results showed that MSCs expressed the surface markers including CD29, CD44 and CD166 and were negative for the expression of CD45, HLA-DR and CD14 (Fig. 1C), indicating the successful isolation of MSCs with the property of multidifferentiation. Subsequently, we extracted and purified sEV from the conditioned medium of MSCs or HFL1. MSC-sEV displayed typical spheroid shapes with the diameter of 171.3 ± 71.7 nm, expressed the protein markers including CD9, CD63, HSP70, Alix, TSG101 and negatively expressed Calnexin (Fig. 1D–F). Similarly, the results of TEM, NTA and western blot demonstrated that HFL1-sEV showed the classical vesicle structure with the diameter of 153.5 ± 62.5 nm and expressed the surface markers (Fig. 1G-I).

### Intravitreal injection of MSC-sEV alleviated retinal apoptosis and oxidative stress in DR rats

**Fig. 2:**
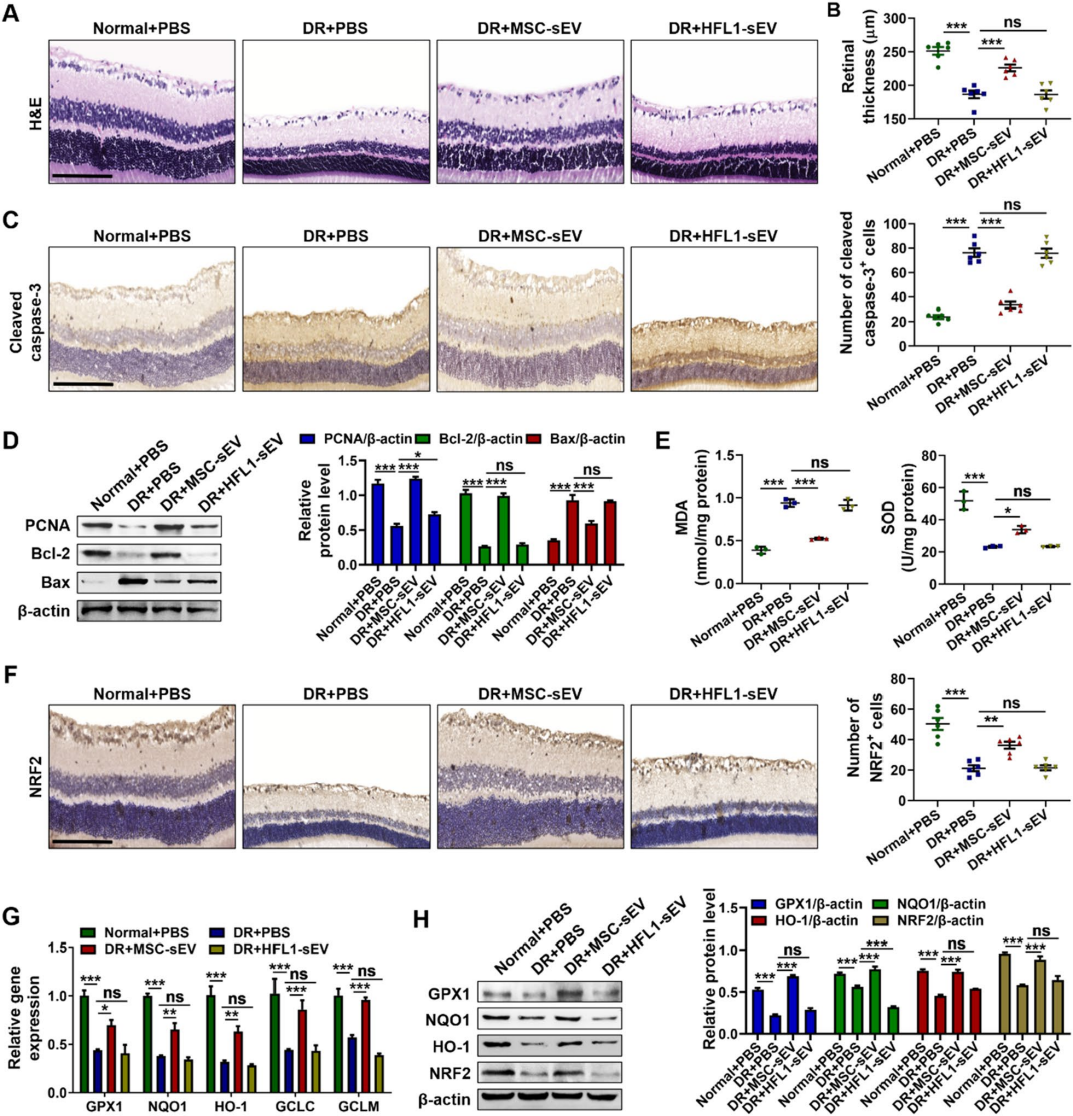
Retinal therapeutic effects of MSC-sEV on diabetic rats. **A** Representative retinal H&E staining images after treatment. Scale bars, 100 μm. **B** Retinal thickness analysis of each group (*n* = 6). **C** Representative images of immunohistochemistry staining of cleaved caspase-3. Scale bars, 100 μm. **D** Western blot analysis for the retinal expression of PCNA, Bcl-2 and Bax. **E** Retinal MDA and SOD levels measurement (*n* = 3). **F** Representative images of retinal immunohistochemistry staining of NRF2. Scale bars, 100 μm. **G** QRT-PCR for the relative mRNA levels of NRF2-related genes (*n* = 3). **H** Western blot analysis for the retinal expression of GPX1, NQO1, HO-1 and NRF2. All data are presented as means ± SEM. ns, not significant, ^*^P < 0.05, ^**^P < 0.01 and ^***^P < 0.001

To investigate whether MSC-sEV serve as an effective therapeutic for DR, we used STZ to establish a rat model of diabetes, followed by the intravitreal injection of PBS, MSC-sEV or HFL1-sEV. As control, HFL1-sEV were used to determine whether MSC-sEV possess the specific ability to alleviate retinal injury. In the scotopic ERG response, the diabetic rats displayed reduced amplitudes of a-wave and b-wave compared with normal rats, while MSC-sEV treatment significantly ameliorated the attenuation of a-wave and b-wave amplitudes, indicating that MSC-sEV improved the diabetes-induced retinal dysfunction and visual decline (Additional file 1: Fig. S1A–C). Retinal H&E staining showed that diabetes promoted the reduction of retinal thickness, whereas MSC-sEV but not HFL1-sEV treatment increased retinal thickness and normalized the retinal morphology (Fig. 2A, B). Immunohistochemistry staining revealed that MSC-sEV decreased the number of cleaved caspase-3 positive cells in retinal tissues (Fig. 2C). Western blot results further confirmed the increased retinal apoptosis level in DR + PBS group, while administration of MSC-sEV downregulated Bax expression and upregulated proliferating cell nuclear antigen (PCNA) and Bcl-2 expressions (Fig. 2D).

Long-term diabetic microenvironment induced the oxidative damage is the main cause of retinal cells loss. Compared with PBS group, treatment with MSC-sEV but not HFL1-sEV significantly reduced malondialdehyde (MDA) level and enhanced superoxide dismutase (SOD) level in the retinas of DR rats (Fig. 2E). NRF2 is widely recognized as an essential regulator of endogenous antioxidant genes [18]. Thus, we analyzed the retinal expression of NRF2 and found that NRF2 and its dependent genes including glutathione peroxide 1 (GPX1), NADPH quinone oxidoreductase 1 (NQO1), heme oxygenase-1 (HO-1), glutamyl cysteine ligase catalytic subunit (GCLC) and glutamate cysteine ligase modifier subunit (GCLM) were notably decreased in PBS-treated retinal tissues of diabetic rats, whereas MSC-sEV treatment restored their expressions (Fig. 2F–H). Taken together, these data indicated that MSC-sEV could relieve retinal apoptosis and oxidative stress to prevent DR progress.

### MSC-sEV protected RPE cells against HG-triggered apoptosis and oxidative damage

**Fig. 3:**
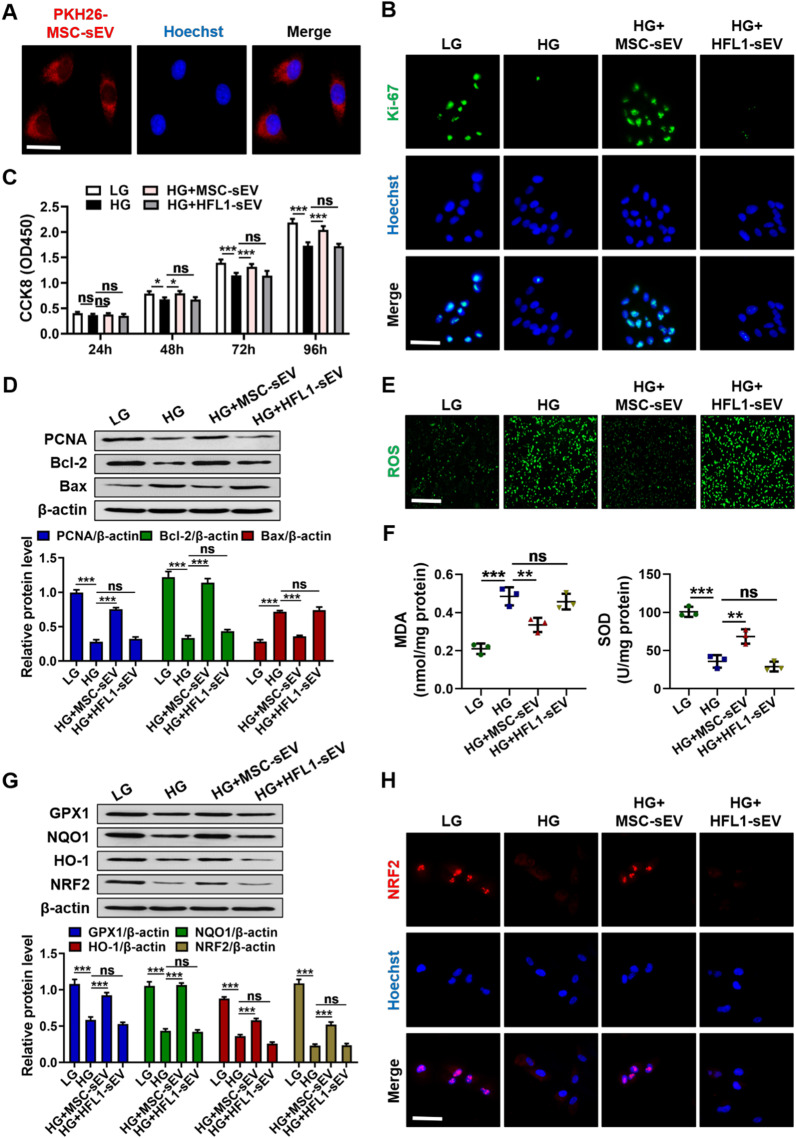
MSC-sEV protected RPE cells against HG conditions in vitro. **A** The internalization of PKH-26-labeled MSC-sEV by RPE cells was detected by a confocal microscope. Scale bars, 25 μm. **B** Immunofluorescence analysis of Ki-67 in RPE cells. Scale bars, 25 μm. **C** CCK8 assay for the proliferation ability of RPE cells (*n* = 4). **D** Western blot analysis for the expressions of PCNA, Bcl-2 and Bax in RPE cells. **E** Detection of ROS generation in RPE cells. Scale bars, 100 μm. **F** MDA and SOD level measurement in RPE cells (*n* = 3). **G** Western blot analysis for the expressions of GPX1, NQO1, HO-1 and NRF2 in RPE cells. **H** Immunofluorescence staining of NRF2 in RPE cells. Scale bars, 25 μm. All data are presented as means ± SEM. ns, not significant, ^*^P < 0.05, ^**^P < 0.01 and ^***^P < 0.001

Due to the critical role of RPE cells in maintaining the retinal homeostasis, we treated RPE cells with HG medium for 48 h to mimic diabetic stimulation in vivo and then added PBS, MSC-sEV or HFL1-sEV to investigate their repairing effects and the potential mechanism. Immunofluorescence staining result showed that PKH-26-labeled MSC-sEV could be internalized by RPE cells after co-incubation (Fig. 3A). Treatment with MSC-sEV but not HFL1-sEV enhanced the expression of Ki-67 in RPE cells (Fig. 3B). Compared to the PBS group, MSC-sEV promoted the proliferation of RPE cells cultured in HG stress, while HFL1-sEV had little effect (Fig. 3C). Western blot analysis of proliferation and apoptosis-associated proteins indicated that the expressions of PCNA and Bcl-2 were higher and the expression of Bax was lower in MSC-sEV group than those in PBS or HFL1-sEV group (Fig. 3D). Subsequently, HG-induced oxidative stress level of RPE cells after different treatments was measured. We found that compared with PBS or HFL1-sEV, MSC-sEV significantly decreased ROS production, reduced MDA level and enhanced SOD expression in HG medium-cultured RPE cells (Fig. 3E, F). NRF2 gene was also downregulated in HG-injured RPE cells, whereas MSC-sEV treatment induced the nuclear translocation of NRF2 and increased its downstream genes expressions including GPX1, NQO1 and HO-1 (Fig. 3G, H). These results suggested that MSC-sEV exerted antioxidant effects to enhance RPE cells viability.

### MSC-sEV exerted retinal therapeutic effects by inhibiting PTEN expression

**Fig. 4:**
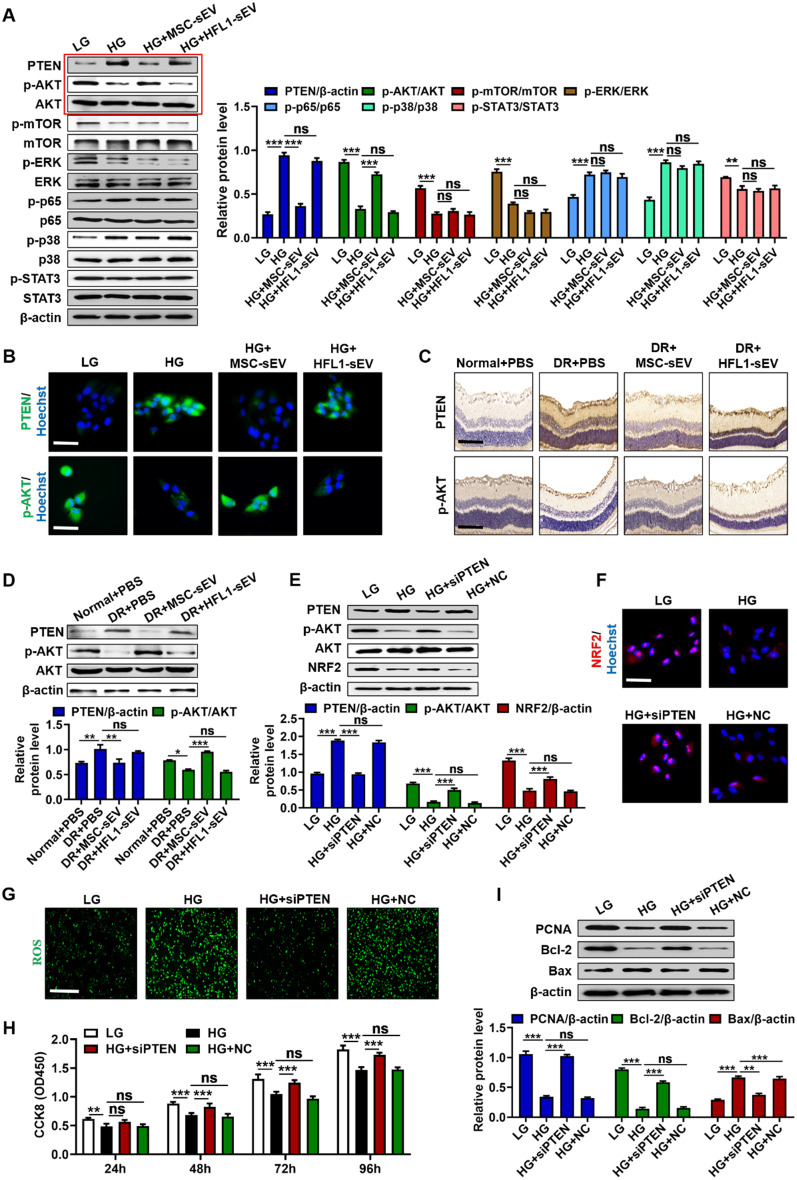
MSC-sEV exerted repairing effects through PTEN/AKT signaling pathway. **A** Western blot analysis for the expressions of cell signaling pathway in RPE cells after treatment. **B** Immunofluorescence staining of PTEN and p-AKT in RPE cells after treatment. Scale bars, 25 μm. **C** Representative immunohistochemistry staining images of PTEN and p-AKT in retinal tissues. Scale bars, 100 μm. **D** Western blot analysis for the expressions of PTEN and p-AKT in retinal tissues. **E** Western blot analysis for the expressions of PTEN, p-AKT and NRF2 in RPE cells transfected with PTEN siRNA. **F** Representative immunofluorescence staining images of NRF2 in RPE cells transfected with PTEN siRNA. Scale bars, 25 μm. **G** Detection of ROS generation in RPE cells transfected with PTEN siRNA. Scale bars, 100 μm. **H** CCK8 assay for the proliferation ability of RPE cells transfected with PTEN siRNA (*n* = 4). **I** Western blot analysis for the expressions of PCNA, Bcl-2 and Bax in RPE cells transfected with PTEN siRNA. All data are presented as means ± SEM. ns, not significant, ^*^P < 0.05, ^**^P < 0.01 and ^***^P < 0.001

To clarify the mechanism for the therapeutic effects of MSC-sEV on retinal damage, we detected the response of several cell signaling pathways in HG condition-cultured RPE cells after MSC-sEV treatment. Western blot analysis demonstrated that MSC-sEV but not HFL1-sEV inhibited PTEN expression and promoted AKT phosphorylation, while there was no significant change in other pathways including mTOR, ERK, p65, p38 and STAT3 after MSC-sEV treatment (Fig. 4A). Immunofluorescent staining results further confirmed MSC-sEV induced the downregulation of PTEN and the upregulation of phosphorylated AKT level in RPE cells under HG stress (Fig. 4B). In consistent with in vitro results, we also observed that intravitreal injection of MSC-sEV reversed hyperglycemia caused the activation of PTEN and increased p-AKT level in retinal tissues of diabetic rats (Fig. 4C, D), suggesting that PTEN/AKT signaling may be involved in the MSC-sEV-mediated protection against DR. Next, we transfected PTEN siRNA into RPE cells to determine the importance of PTEN in DR progress. Western blot results showed that the knockdown of PTEN significantly promoted AKT phosphorylation and increased NRF2 expression (Fig. 4E, F). The reduced production of ROS was also detected after PTEN siRNA transfection (Fig. 4G). Furthermore, PTEN knockdown enhanced the proliferation activity of RPE cells (Fig. 4H). HG stress caused the increased expression of Bax and the inhibition of PCNA and Bcl-2 were also reversed by PTEN knockdown (Fig. 4I). Thus, the above results revealed that PTEN was an important therapeutic target of DR.

### PTEN/AKT axis was responsible for MSC-sEV-mediated retinal protection

**Fig. 5:**
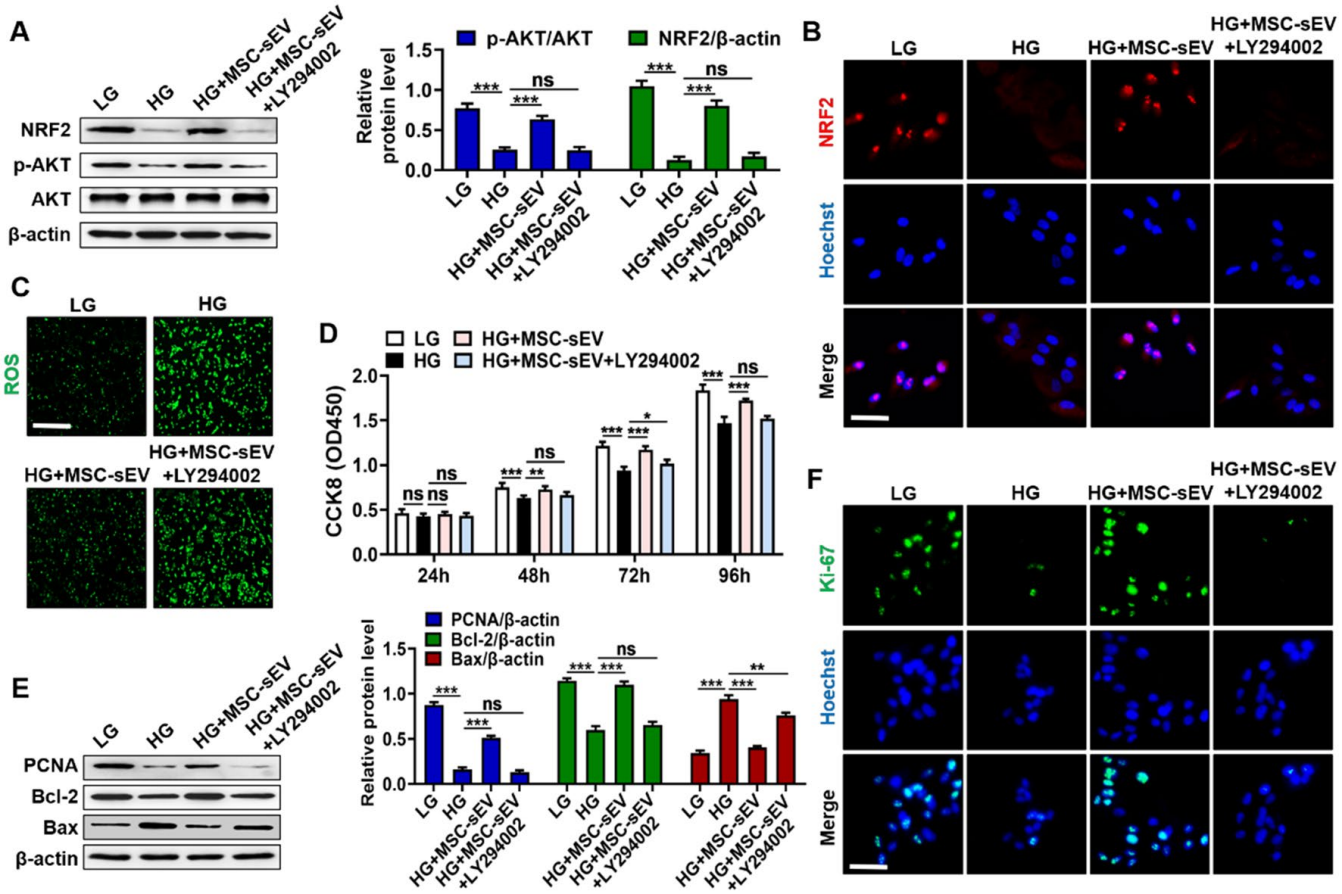
AKT inhibition impaired MSC-sEV-induced therapeutic effects in vitro. **A** Western blot analysis for the expressions of NRF2 and p-AKT in RPE cells treated with LY294002. **B** Immunofluorescence staining of NRF2 in RPE cells treated with LY294002. Scale bars, 25 μm. **C** Detection of ROS generation in RPE cells treated with LY294002. Scale bars, 100 μm. **D** CCK8 assay for the proliferation ability of RPE cells treated with LY294002 (*n* = 4). **E** Western blot analysis for the expressions of PCNA, Bcl-2 and Bax in RPE cells treated with LY294002. **F** Representative immunofluorescence staining images of Ki-67 in RPE cells treated with LY294002. Scale bars, 25 μm. All data are presented as means ± SEM. ns, not significant, *P < 0.05, **P < 0.01 and ***P < 0.001

To illustrate whether MSC-sEV-induced PTEN inhibition contributes to the retinal protection by regulating AKT activation, we treated RPE cells with MSC-sEV and AKT inhibitor LY294002 simultaneously. The increased expression of NRF2 and the reduced generation of ROS after MSC-sEV treatment could be abolished by LY294002 (Fig. 5A–C). We further detected the importance of AKT signaling pathway in MSC-sEV induced the improvement of cell vitality. The result of CCK8 assay showed that MSC-sEV could promote RPE cells proliferation, which were partially reversed by AKT inhibition (Fig. 5D). Compared to MSC-sEV group, the simultaneous treatment with LY294002 reduced the protein levels of PCNA and Bcl-2 and enhanced the expression of Bax (Fig. 5E). Moreover, AKT inhibition reversed MSC-sEV mediated the increased expression of Ki-67 in RPE cells (Fig. 5F). These findings pointed out that MSC-sEV induced the downregulation of PTEN prevented RPE cells from HG stress mainly through the activation of AKT signaling.

### MSC-sEV delivered NEDD4 to promote PTEN degradation

**Fig. 6:**
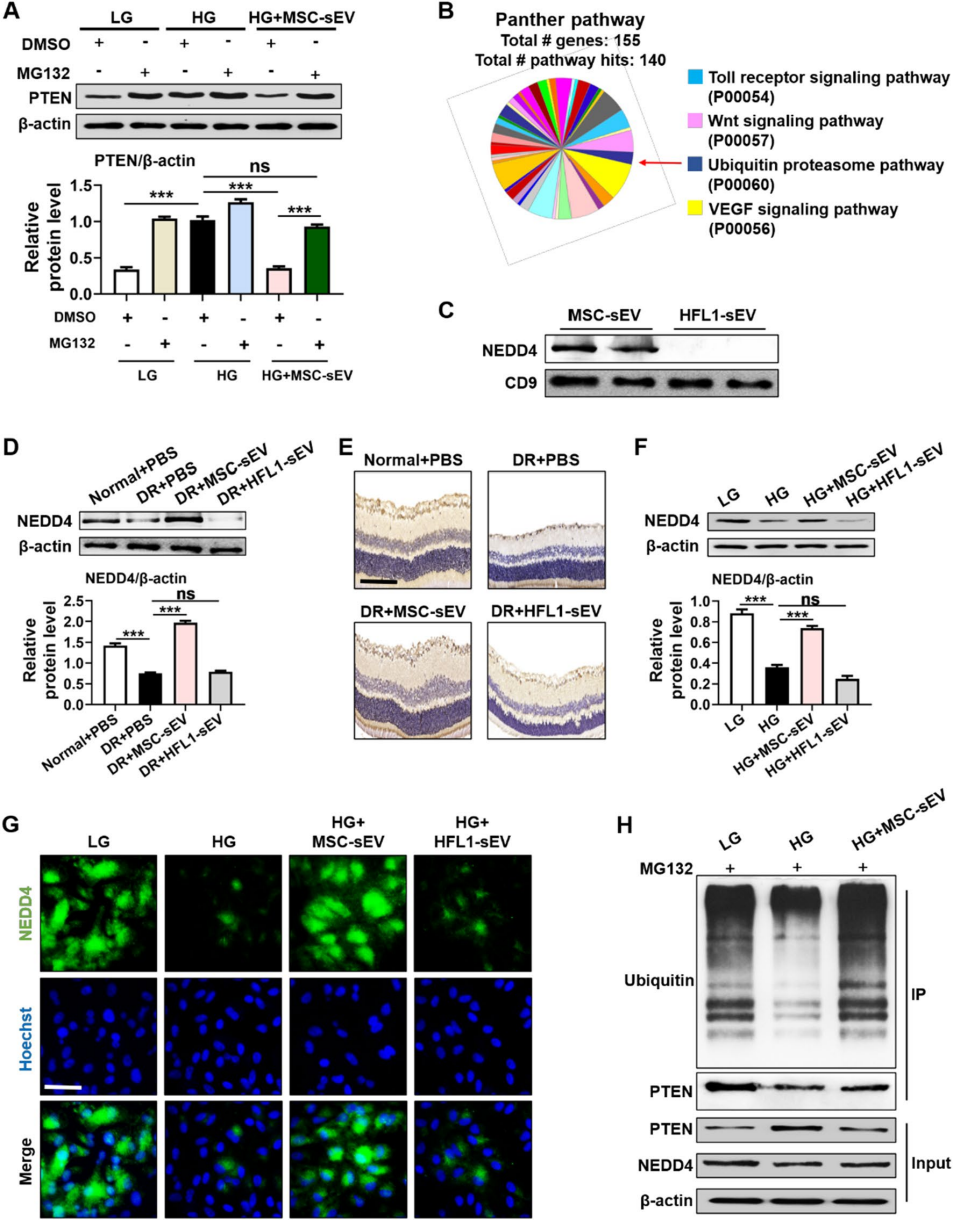
MSC-sEV-delivered NEDD4 promoted PTEN ubiquitination and degradation. **A** Western blot analysis for the expression of NEDD4 in RPE cells treated with MG132. **B** Mass spectrometry analysis results of MSC-sEV protein content. **C** Western blot analysis for the expression of NEDD4 in MSC-sEV and HFL1-sEV. **D** Representative immunohistochemistry staining images of NEDD4 in retinal tissues after treatment. Scale bars, 100 μm. **E** Western blot analysis for the expression of NEDD4 in retinal tissues after treatment. **F** Western blot analysis for the expression of NEDD4 in RPE cells after treatment. **G** Representative immunofluorescence staining images of NEDD4 in RPE cells after treatment. Scale bars, 25 μm. **H** The ubiquitination of PTEN protein after MSC-sEV treatment and the binding of NEDD4 and PTEN were detected by co-IP assay. All data are presented as means ± SEM. ns, not significant, ^***^P < 0.001

Next, we explored the key molecules in MSC-sEV to mediate the inhibition of PTEN and retinal protective effects. MG132 assay was firstly performed to determine whether MSC-sEV regulate PTEN protein stability. Western blot results demonstrated that pre-treatment with MG132 restored MSC-sEV induced the decreased level of PTEN in RPE cells (Fig. 6A), implying that MSC-sEV reduce PTEN expression mainly through ubiquitin–proteasome pathway. We then carried out mass spectrometry to analyze the protein profile of MSC-sEV. The ubiquitin–proteasome system-associated proteins were identified to be abundant in MSC-sEV (Fig. 6B). Previous studies have shown that NEDD4-mediated ubiquitination is a major pathway for PTEN degradation [19]. To this end, we verified the expression of NEDD4 in MSC-sEV (Fig. 6C). Subsequently, the expression of NEDD4 was measured in the retinal tissues of diabetic rats. We found that MSC-sEV treatment reversed hyperglycemia induced the decreased NEDD4 expression in vivo (Fig. 6D, E). Meanwhile, HG stimulation significantly inhibited the activation of NEDD4, whereas MSC-sEV but not HFL1-sEV enhanced NEDD4 protein level in RPE cells (Fig. 6F, G). The result of co-IP further revealed that MSC-sEV-delivered NEDD4 could combine with PTEN and enhance PTEN ubiquitinated modification (Fig. 6H). These results indicated that MSC-sEV promoted PTEN ubiquitination and degradation by transporting NEDD4, thus exerting retinal therapeutic effects.

### NEDD4 knockdown impaired the therapeutic effects of MSC-sEV on retinal injury

To evaluate the importance of NEDD4 in the retinal repairing effects of MSC-sEV, we knocked down NEDD4 expression in MSCs by lentiviral vector-mediated shRNA transfection and then isolated sEV from NEDD4 shRNA-transfected MSCs. Western blot results showed that the expression of NEDD4 was significantly decreased in MSC^shNEDD4^ and MSC-sEV^shNEDD4^ groups compared with control (Additional file 2: Fig. S2A, B). Subsequently, diabetic rats were injected with PBS, MSC-sEV^shGFP^ or MSC-sEV^shNEDD4^ intravitreally. ERG analysis demonstrated that the amplitudes of a-wave and b-wave were reduced in MSC-sEV^shNEDD4^ group compared with MSC-sEV^shGFP^ group (Additional file 2: Fig. S2C–E). NEDD4 knockdown suppressed MSC-sEV mediated the preservation of retinal integrity and thickness (Additional file 2: Fig. S2F). The decreased expression of NEDD4 also prevented MSC-sEV to alleviate hyperglycemia-induced retinal apoptosis (Additional file 2: Fig. S2G, H). Moreover, we assessed the retinal oxidative damage of diabetic rats after different treatments. MSC-sEV^shNEDD4^ injection had little effect to downregulate MDA level and enhance SOD level in the retinas of diabetic rats (Additional file 2: Fig. S2I, J). Furthermore, western blot and immunohistochemistry staining results revealed that NEDD4 knockdown weakened MSC-sEV induced the upregulation of NRF2-related genes (Additional file 2: Fig. S2K, L). These results indicated that NEDD4 was critical for the retinal therapeutic effects of MSC-sEV.

## Discussion

Retinal degeneration represents one significant cause of vision decline and incurable sight impairment. RPE cells are reported to maintain retinal structure and function through protecting other retinal cells against malignant microenvironments [20, 21]. Increasing studies reveal that RPE cells are susceptible to oxidative damage caused by diabetic conditions [22]. RPE cells apoptosis is recognized as one major pathogenesis of retinal damage in DR with limited therapeutic strategies. Recently, MSCs transplantation to improve RPE cells regeneration has achieved encouraging progress [23]. Through replacing injured cells, MSCs exert profound therapeutic effects to mediate retinal recovery. However, the evidence displaying the intravitreal injection of MSCs-induced serious ocular complications suggests that the security of cell-based therapy needs further investigations [24]. As the major repairing mechanism underlying MSCs transplantation, sEV carrying various bioactive molecules are identified as an alternative approach to avoid the existing defects and retain the protective effects of MSCs treatment [25]. Although the potential benefits of MSC-sEV to protect RGC cells, photoreceptors and retinal vascular endothelial cells against pathological environment have been reported [26–28], it is unclear whether MSC-sEV exhibit positive actions to injured RPE cells exposed to HG medium. In this study, we revealed that MSC-sEV efficiently counteracted hyperglycemia-induced retinal disorder by alleviating the oxidative injury and apoptosis. Notably, the single intravitreal injection of MSC-sEV exerted a sustained retinal protection in the STZ-induced diabetic model, thus offering possibility for establishing effective approach to treat DR.

Increased retinal oxidative stress and apoptosis are critical features in the early stage of DR [29]. NRF2 is a major transcription factor which regulates cellular antioxidant signaling and promotes cell survival, therefore ameliorating numerous diseases [30]. Through nuclear translocation, NRF2 interacts with antioxidant responsive elements and activates the downstream antioxidant genes, including GPX1, NQO1 and HO-1 [31]. During DR development, the decreased NRF2 level significantly contributes to retinal oxidative damage [32]. Through in vivo and in vitro experiments, we confirmed the downregulation of NRF2 and NRF2-related genes under diabetic conditions. Our findings further illustrated that MSC-sEV-mediated nuclear accumulation of NRF2 reduced ROS production and alleviated retinal cells loss.

Emerging studies have revealed that PTEN is involved in the regulation of multiple cellular signal transductions and biological functions such as cell proliferation, growth and apoptosis [33]. Abnormal PTEN level contributes to the progress of various diseases including retinal degeneration. Kim et al. suggest that PTEN deficiency causes the ocular metabolism disorder of retinoic acids [34]. DR-induced PTEN overexpression promotes retinal neuronal cells death [35]. RGCs loss-mediated optic nerve injury is closely associated with the increase in PTEN [36]. Importantly, PTEN is widely proven as an antagonist of the AKT signaling pathway, which can upregulate NRF2 expression to alleviate oxidative damage and ROS release [37]. Herein, we found that MSC-sEV inhibited HG conditions induced the activation of PTEN and promoted AKT phosphorylation, thus upregulating NRF2 expression. PTEN knockdown significantly enhanced the proliferation and antioxidative abilities of RPE cells, supporting the notion that PTEN is a key target for DR therapy. Moreover, LY294002 treatment impaired MSC-sEV-mediated therapeutic effects, further confirming that PTEN/AKT signaling pathway is essential for the roles of MSC-sEV.

Given the importance of sEV in intercellular communication, there are numerous studies exploring the composition of sEV. It is reported that the cargos in sEV mainly divide into proteins, lipids and nucleic acids [38]. Shuttle of active proteins has been widely considered as a significant pathway for MSC-sEV to promote tissue regeneration [39]. As an important member of HECT domain E3 ubiquitin ligase family, NEDD4 plays a critical role in several pathophysiological processes. Downregulation of NEDD4 results in the embryonic death and neurite growth restriction [40, 41]. Moreover, Monami et al. also suggest that NEDD4 is involved in the insulin signaling transfer and insulin growth factor-1 expression [42]. Accumulating evidence shows that NEDD4 contributes to the degradation of PTEN through the ubiquitin–proteasome pathway [43]. In this study, our results showed that MSC-sEV inhibited the HG conditions-induced activation of PTEN. To identify the mechanism underlying the regulation of MSC-sEV on PTEN expression, we performed MG132 assay and found that MG132 treatment prevented MSC-sEV-mediated downregulation of PTEN, suggesting that MSC-sEV accelerate PTEN degradation mainly through ubiquitin–proteasome pathway. Through mass spectrometry analysis, we for the first time found that NEDD4, a central ubiquitination-related enzyme for PTEN degradation, was abundant in MSC-sEV. Increased NEDD4 expression could be observed after MSC-sEV administration. Importantly, NEDD4 knockdown significantly impaired MSC-sEV-induced retinal therapeutic effects, suggesting that MSC-sEV-delivered NEDD4 was crucial for the retinal protection.

## Conclusion

**Fig. 7:**
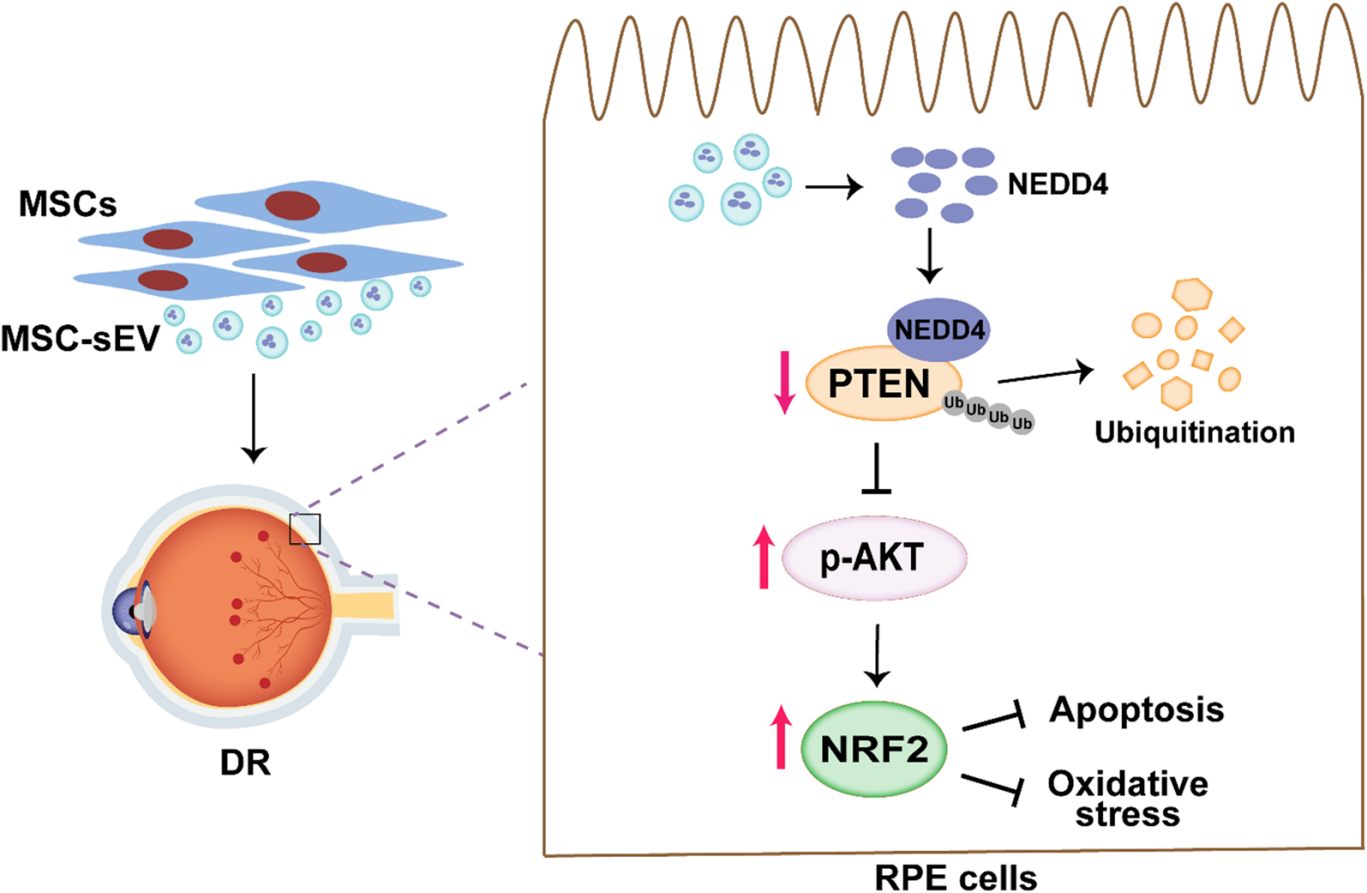
Proposed model for the therapeutic roles of MSC-sEV in DR. MSC-sEV-delivered NEDD4 mediates the ubiquitination and degradation of PTEN, thus promoting the activation of AKT signaling pathway and upregulating NRF2 expression to alleviate the oxidative damage and apoptosis of RPE cells

In summary, the results of the present study revealed that MSC-sEV treatment effectively alleviated retinal oxidative stress and apoptosis to prevent DR progress through PTEN/AKT/NRF2 signaling pathway. MSC-sEV-delivered NEDD4 promoted the ubiquitination and degradation of PTEN, thus activating AKT phosphorylation and upregulating NRF2 expression (Fig. 7). Our findings expand the understanding of DR pathogenesis and provide promise for establishing a novel strategy for DR therapy.

## Supplementary Information

**Additional file 1: Figure.S1**. MSC-sEV improved retinal function in diabetic rats.

**Additional file 2: Figure. S2**. NEDD4 knockdown impaired MSC-sEV-induced retinal therapeutic effects in vivo.

## Data Availability

The data used to support the findings of this study are available from the corresponding author upon request.

## Abbreviations

BSA: Bovine serum albumin
CCK8: Cell counting kit-8
Co-IP: Co-immunoprecipitation
DMEM: Dulbecco’s modified Eagle’s medium
DR: Diabetic retinopathy
ERG: Electroretinography
FBS: Fetal bovine serum
GCLC: Glutamyl cysteine ligase catalytic subunit
GCLM: Glutamate cysteine ligase modifier subunit
GPX1: Glutathione peroxide 1
HFL1: Human fetal lung fibroblast 1
HG: High glucose
HFL1-sEV: Human fetal lung fibroblast 1-derived small extracellular vesicles
HO-1: Heme oxygenase-1
HSP70: Heat shock protein 70
H&E: Hematoxylin and eosin
LG: Low glucose
MDA: Malondialdehyde
MSCs: Mesenchymal stem cells
MSC-sEV: Mesenchymal stem cells-derived small extracellular vesicles
NEDD4: Neuronal precursor cell-expressed developmentally downregulated 4
NQO1: NADPH quinone oxidoreductase 1
NRF2: Nuclear factor erythroid 2-related factor 2
NTA: Nanoparticle tracking analysis
PBS: Phosphate-buffered saline
PCNA: Proliferating cell nuclear antigen
PTEN: Phosphatase and tensin homolog
ROS: Reactive oxygen species
sEV: Small extracellular vesicles
SOD: Superoxide dismutase
STZ: Streptozotocin
TEM: Transmission electron microscopy
TSG101: Tumor susceptibility gene 101
α-MEM: Minimal essential medium alpha
