# Supplementary material for: Mesenchymal stem cells-derived small extracellular vesicles alleviate diabetic retinopathy by delivering NEDD4
Source: Stem Cell Res Ther. 2022 Jul 15;13:293. doi: 10.1186/s13287-022-02983-0 (PMC9284871; doi:10.1186/s13287-022-02983-0)
Supplement: Supplementary file 1 — Additional file 1: Figure.S1. MSC-sEV improved retinal function in diabetic rats. [file 13287_2022_2983_MOESM1_ESM.docx]

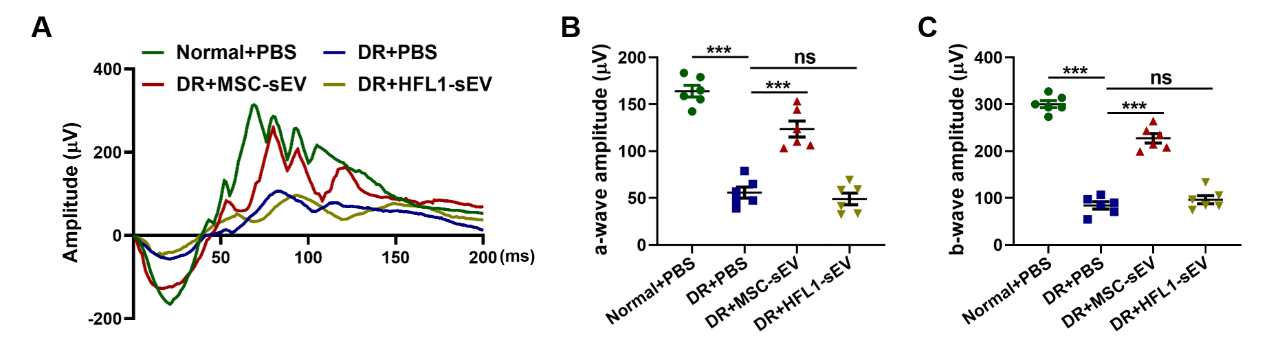


**Additional file 1: Figure S1.** MSC-sEV improved retinal function in diabetic rats. (A) Representative scotopic ERG waveforms of each group. (B) Quantitative analysis of amplitude changes of a-wave (n=6). (C) Quantitative analysis of amplitude changes of b-wave (n=6). All data are presented as means ± SEM. ns, not significant, ^***^*P*<0.001.
