# Supplementary material for: Mesenchymal stem cells-derived small extracellular vesicles alleviate diabetic retinopathy by delivering NEDD4
Source: Stem Cell Res Ther. 2022 Jul 15;13:293. doi: 10.1186/s13287-022-02983-0 (PMC9284871; doi:10.1186/s13287-022-02983-0)
Supplement: Supplementary file 2 — Additional file 2: Figure. S2. NEDD4 knockdown impaired MSC-sEV-induced retinal therapeutic effects in vivo. [file 13287_2022_2983_MOESM2_ESM.docx]

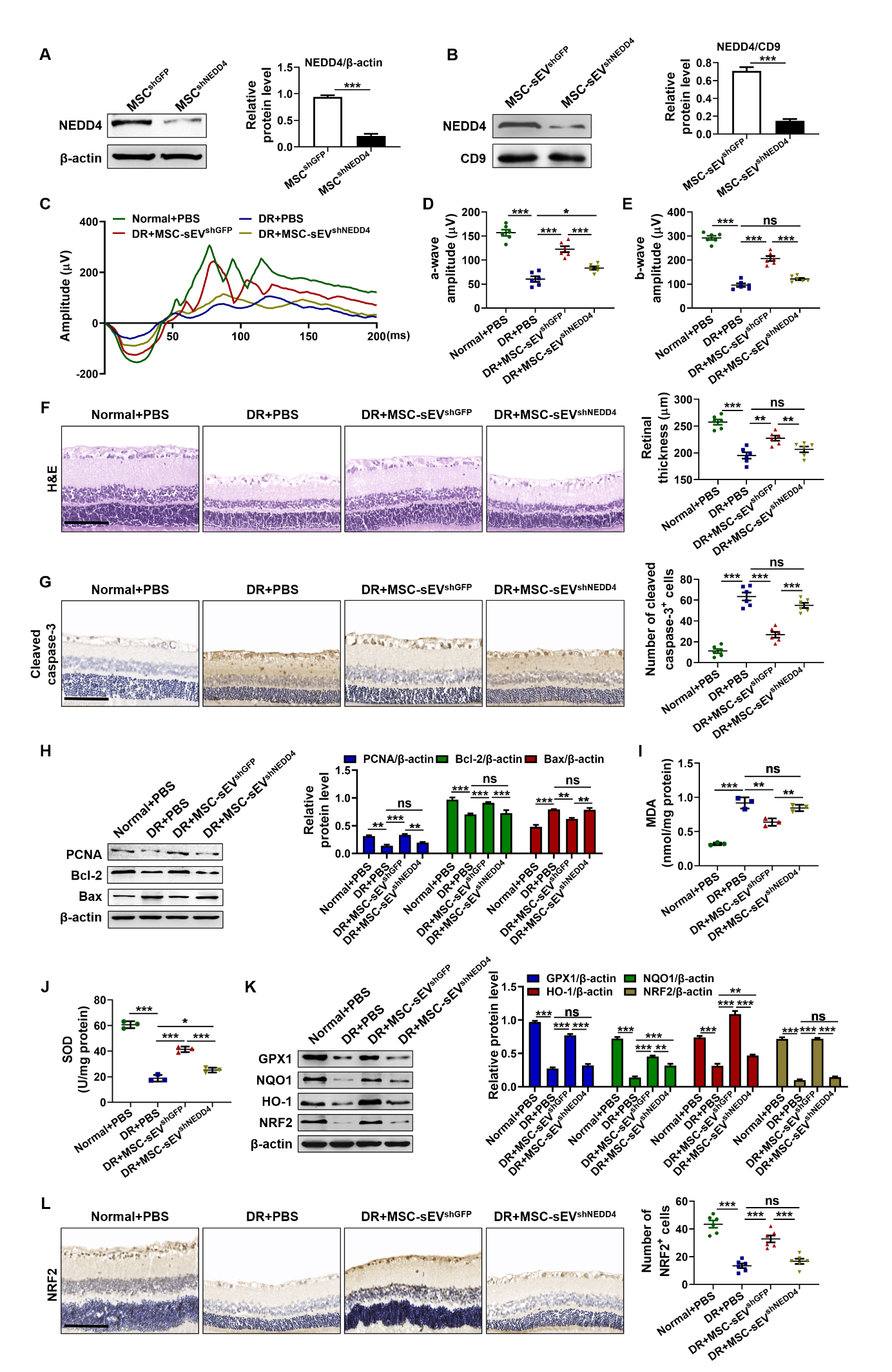


**Additional file 2: Figure S2.** NEDD4 knockdown impaired MSC-sEV-induced retinal therapeutic effects in vivo. (A) Western blot analysis for the expression of NEDD4 in MSCs transfected with NEDD4 shRNA. (B) Western blot analysis for the expression of NEDD4 in MSC-sEV. (C) Representative scotopic ERG waveforms of rats injected with PBS, MSC-sEV^shGFP^ or MSC-sEV^shNEDD4^. (D) Quantitative analysis of amplitude changes of a-wave (n=6). (E) Quantitative analysis of amplitude changes of b-wave (n=6). (F) Representative retinal H&E staining images after treatment and retinal thickness analysis (n=6). Scale bars, 100 μm. (G) Representative images of immunohistochemistry staining of cleaved caspase-3. Scale bars, 100 μm. (H) Western blot analysis for the retinal expression of PCNA, Bcl-2 and Bax. (I) Retinal MDA level measurement (n=3). (J) Retinal SOD level measurement (n=3). (K) Western blot analysis for the retinal expression of GPX1, NQO1, HO-1 and NRF2. (L) Representative images of retinal immunohistochemistry staining of NRF2. Scale bars, 100 μm. All data are presented as means ± SEM. ns, not significant, ^*^*P*<0.05, ^**^*P*<0.01 and ^***^*P*<0.001.
